# Psychotherapies for PTSD: what do they have in common?

**DOI:** 10.3402/ejpt.v6.28186

**Published:** 2015-08-14

**Authors:** Ulrich Schnyder, Anke Ehlers, Thomas Elbert, Edna B. Foa, Berthold P. R. Gersons, Patricia A. Resick, Francine Shapiro, Marylène Cloitre

**Affiliations:** 1 Department of Psychiatry and Psychotherapy, University Hospital Zurich, University of Zurich, Switzerland; 2 Department of Experimental Psychology, University of Oxford, Oxford, UK; 3 Department of Psychology, University of Konstanz, Konstanz, Germany; 4 Center for the Treatment and Study of Anxiety, Department of Psychiatry, University of Pennsylvania, Philadelphia, PA, USA; 5 Academic Medical Center, University of Amsterdam, Diemen, The Netherlands; 6 Department of Psychiatry and Behavioral Sciences, Division of Translational Science, Duke University Medical Center, Durham, NC, USA; 7 Mental Research Institute, Palo Alto, CA, USA; 8 National Center for PTSD—Dissemination & Training Division, VA Palo Alto Health Care System, Palo Alto, CA, USA; 9 NYU Langone Medical Center, New York City, NY, USA

**Keywords:** Psychotraumatology, posttraumatic stress disorder, complex PTSD, psychotherapy, exposure, cognitive restructuring, psychoeducation

## Abstract

Over the past three decades, research and clinical practice related to the field of traumatic stress have developed tremendously. In parallel with the steady accumulation of basic knowledge, therapeutic approaches have been developed to treat people suffering from posttraumatic stress disorder (PTSD) and other trauma-related psychological problems. Today, a number of evidence-based treatments are available. They differ in various ways; however, they also have a number of commonalities. Given this situation, clinicians may wonder which treatment program to use, or more specifically, which treatment components are critical for a successful therapy. In this article, seven pioneers who have developed empirically supported psychotherapies for trauma-related disorders were asked to compose an essay of three parts: first, to provide a brief summary of the treatment they have developed; second, to identify three key interventions that are common and critical in treating PTSD; and third, to suggest important topics and future directions for research. The paper ends with a summary highlighting the identified commonalities (psychoeducation; emotion regulation and coping skills; imaginal exposure; cognitive processing, restructuring, and/or meaning making; emotions; and memory processes), pointing to future directions such as trying to better understand the underlying mechanisms of action, and developing treatments that are tailored to the needs of different patient groups.

Over the past 3 decades, the field of traumatic stress–related research and clinical practice has developed tremendously. In parallel with the steady accumulation of basic knowledge, therapeutic approaches have been developed to treat people suffering from posttraumatic stress disorder (PTSD) and other trauma-related psychological problems. Today, a number of evidence-based treatments are available (Bisson, Roberts, Andrew, Cooper, & Lewis, 2013; Bradley, Greene, Russ, Dutra, & Westen, 2005; Schnyder & Cloitre, 2015; Watts et al., 2013). They differ in various ways including the duration and number of sessions as well as the number and diversity of interventions. Some treatment programs focus on *in vivo* exposure to threat stimuli, whereas others concentrate on the reappraisal of the event without requiring direct confrontation of threat-related stimuli. Strategies for the “processing” of the traumatic memory differ, with some therapies promoting recounting of the trauma via a verbal report, others prescribing a written narrative, and still others including continual or intermittent imagining and experiencing of the traumatic event without verbalization. Some therapies incorporate coping skills at the beginning of the treatment, others integrate them during the course of the treatment, and still others do not include explicit attention to skills building. Some therapies look at trauma across the life span, thus aiming to create a coherent autobiographical narrative, whereas others focus exclusively on a single traumatic event. Some try to integrate traumatic memories with other, more positive life events, others do not.

Given this diversity, clinicians may wonder which treatment program to use, or more specifically, which treatment components are critical for a successful therapy. At the 2014 annual meeting of the International Society for Traumatic Stress Studies in Miami, Drs. Schnyder and Cloitre organized a panel of pioneers who have developed empirically supported psychotherapies, and proposed a challenging task, namely to identify and discuss commonalities across the treatments. Ultimately, the discussion evolved to asking each panel member to identify the three most important interventions for successful trauma therapy.^1^

The purpose of this paper is to provide an answer to that question. In this article, seven pioneers who have developed empirically supported psychotherapies for trauma-related disorders were asked to compose (in alphabetical order of their family names) an essay of three parts: first, to provide a brief summary of the treatment they have developed; second, to identify three key interventions that are common and critical in treating PTSD; and third, to suggest important topics and future directions for research. Every discipline, acknowledged as early as Darwin, has its “lumpers and splitters” (Endersby, 2009). A “lumper” is someone who organizes phenomena in a way that takes the gestalt view and assumes that differences are not as important as signature similarities. A “splitter,” by contrast, creates precise definitions and emphasizes differences over similarities. Psychotherapy research may be dominated by this perspective, at least when it proceeds with the experimental goal of identifying and systematically testing potential “active ingredients” within a treatment (e.g., component analysis).

We have, therefore, asked the coauthors of this article to abandon what might be the more comfortable mind-set, and to think about the field of psychotherapy for PTSD through a “big picture” perspective, organizing it into broad categories that define critical treatment components, and to discuss implications for important research in the future. Each coauthor's contribution should look forward to the next steps in the field rather than look back and critique it. The following essays were invited as “points of view”. The paper will end with a summary highlighting the identified commonalities, as far as they exist, and pointing to future directions.

## Marylène Cloitre (STAIR Narrative Therapy)

Skills Training in Affective and Interpersonal Regulation (STAIR) Narrative Therapy is an evidence-based two-component therapy that provides training in emotion regulation and social skills in combination with trauma narrative analysis. The impetus for developing this treatment was simple, based on both clinical observation and the empirical literature. Patients often come to treatment motivated by problems in relationships and by emotional disturbances as significant if not primary concerns. Introducing skills training to address these problems at the beginning of treatment provides a therapy that is transparently responsive to and in-sync with the patients’ primary concerns. This approach emphasizes treatment planning according to patient-specific goals, values, and preferences. Not surprisingly, the use of STAIR preceding narrative therapy supports retention in care and results in superior improvement in perceived social support and emotion regulation capacities along with enhanced PTSD reduction relative to trauma exposure without skills training (Cloitre et al., 2010). Trauma-focused treatments for PTSD have been widely disseminated in the United States Veterans Health Administration and have been found to be highly effective for those who complete the treatments. However, several studies have consistently shown that less than 10% of veterans with PTSD do so (Mott, Hundt, Sansgiry, Mignogna, & Cully, 2014; Seal et al., 2010; Watts et al., 2014). Although systems factors may in part explain this slow uptake, it allows consideration of whether the treatments are meeting patient needs and how to deliver treatments that better engage patients.

The above treatment, as well as many other effective trauma therapies, share several features in common and I would propose that the three most important include: *improvement in emotion regulation*, *making meaning* of the traumatic events, and the ubiquitous but powerful *psychoeducation*. The benefit of improvement in emotion regulation is that it allows the individual to feel calm, to engage in goal directed activity, and to develop better relationships and social networks (Hassija & Cloitre, 2013). Although trauma-focused therapies may indirectly improve emotion regulation, changes in this domain are larger, when practices which directly strengthen emotion regulation (e.g., skills training) are included in the treatment (Cloitre et al., 2010). Making meaning typically involves adaptive reappraisal of trauma-generated beliefs about self and others which yields improvement in self-regard, sets a frame that supports greater social engagement, and provides hopefulness and optimism about the future. Psychoeducation is an integral part of skills training and meaning making. It also involves the transmission of basic information such as that trauma is common, its effects are well recognized, and effective interventions are available. Acknowledgement of the reality of trauma, its psychological impact, and the identification of the possibility of recovery provides a sense of support and hope, well-known factors in recovery from and protection against future traumas (Hassija & Cloitre, 2013).

There is quite a range of important next steps (Cloitre, 2015). Patient-centered care is a priority in research and clinical service. Professionals are partnering with patients to identify outcomes that are important to them, treatment strategies that can be easily integrated into daily life, and service models that are efficient and respectful of patients’ time and comfort. Treatment interventions that are responsive to the impact of the cumulative effects of repeated and multiple trauma exposure need to be better articulated and evaluated, with particular attention to the personal and environmental resource losses that occur and erode capacity for recovery (Hobfoll, 2002). The fact that many individuals continue to be exposed to traumatic stressors (e.g., ethnic or community violence) even as they seek treatment indicates the importance of treatment models and strategies that strengthen protective or “resiliency” factors such as social support, community and family bonds, and perceived self-efficacy (Southwick, Bonanno, Masten, Panter-Brick, & Yehuda, 2014). All of the above indicate the importance of supporting innovation in treatment strategies and models. Identification of underlying mechanisms of action (e.g., changes in emotion regulation and cognition) will help to characterize the key elements of effective treatments as innovation in treatment strategies and interventions continues.

## Anke Ehlers (Cognitive Therapy for PTSD)

Cognitive Therapy for PTSD includes five core treatment procedures. First, therapist and patient collaboratively develop *an individualized case formulation*, that is, individualized version of Ehlers and Clark's (Ehlers & Clark, 2000) cognitive model of PTSD, which serves as the framework for therapy. Treatment procedures are tailored to the formulation. Second, *updating trauma memories* is a three-step procedure that includes (1) accessing memories of the worst moments during the trauma and their currently threatening meanings, (2) identifying information that updates these meanings (either information from course of events during the trauma or from cognitive restructuring and testing of predictions), and (3) linking the new meanings to the worst moments in the memory. Third, *discrimination training with triggers of reexperiencing* involves systematically spotting idiosyncratic triggers (often subtle sensory cues) and learning to discriminate between NOW (cues in a new safe context) and THEN (cue in the traumatic situation). Fourth, *dropping unhelpful behaviors and cognitive processes* commonly includes behavioral experiments where the patient experiments with reducing unhelpful strategies such as rumination, hypervigilance for threat, thought suppression, and excessive precautions (safety behaviors) (Ehring, Ehlers, & Glucksman, 2008). Fifth, *reclaiming your life assignments* are designed to address the patients’ perceived permanent change after trauma and involve reclaiming or rebuilding activities and social contacts. The treatment includes elements of the procedures mentioned in the introduction with the exception of teaching *coping strategies* to reduce arousal and distress when a trauma memory is triggered. This is not a routine procedure and is only used for certain presentations, patients with high degrees of dissociation (grounding techniques help the patient stay aware of the “here and now”) or patients with high degrees of anger.

In my view, the *key common elements* of evidence-based psychological treatments for PTSD are best conceptualized in terms of the mechanisms by which they can promote change. Particular treatment techniques can have several functions in therapy. *Psychoeducation*, for example, can be used to motivate patients, create hope, and help address negative appraisals such as misinterpretations of symptoms. Similarly, different techniques may lead to similar changes in candidate mechanisms (e.g., problematic appraisals can be shifted by prolonged exposure or cognitive restructuring).

First, the treatments have in common that they change problematic *meanings* (appraisals) of the trauma about the self and the world. Evidence is emerging that change in appraisals mediates change in PTSD symptoms in a range of evidence-based treatments (e.g., Kleim et al., 2013). A new perspective on problematic meanings may be generated in several ways, (1) considering the trauma and its context in detail, (2) cognitive restructuring and testing predictions in behavioral experiments, or (3) simultaneously bringing to mind old and new meanings.

Second, the treatments access and change the *memory* of the traumatic event. The degree of exposure varies widely, as does the focus on the whole trauma memory versus particular moments. The moments that are reexperienced are especially important to access, as they are usually linked to problematic meanings and can be difficult to access sufficiently by just talking about the trauma, because of, for example, avoidance and the disjointedness of trauma memories. It is interesting to note that in PTSD, these moments appear to retain threatening meanings despite evidence to the contrary. The treatments appear to integrate this evidence into the memory.

Third, the treatments facilitate learning to *discriminate* between the trauma and the present, often by bringing both simultaneously to mind. This can include distinguishing between the trauma and other parts of one's life, and refocusing one's attention on life outside the trauma. It can include patients learning that the triggers of reexperiencing and strong emotions are not harmful in the present context or that the negative aspects of the self that are perceived during the trauma do not apply to their lives in general.

Some future challenges include better understanding of the cumulative effects of repeated or multiple traumas and how best to address them in treatment. Work on how to treat comorbidity most efficiently is also needed. Although progress has been made in predicting who will develop chronic PTSD after trauma and evidence-based treatments have been shown to be effective as early interventions, it remains unclear whether PTSD can be prevented. This question appears especially important for high-risk populations such as military or emergency personnel.

## Thomas Elbert, Maggie Schauer, and Frank Neunert (Narrative Exposure Therapy)

From social exclusion to emotional torment to the consistent wear and tear of living with adversity, stressors not only demand immediate responses but also leave lasting imprints that remodel the systemic functioning of the body, mind, and behavior. Each new episodic threat to life and integrity encountered does not strike a blank canvas but is processed by an individual who has been formed by experiences: Thus, the perception of any emotionally arousing event will be interpreted and categorized based on the memories of previously experienced stressors. With each additional traumatizing experience, the survivor increasingly perceives threats to life and integrity as being omnipresent. For the individual, the context enveloping each cue slowly disappears (Elbert, Schauer, & Neuner, 2015), and without this orienting context, the individual is left to experience the threat without understanding from where it is coming: this is the gateway to PTSD symptoms. Amid the backdrop of the effects of cumulative exposure to stress, Narrative Exposure Therapy (NET), developed by Schauer, Neuner, and Elbert (2011), focuses on the experiences with the strongest arousal responses, especially those evoking fear and helplessness leading to alarm or dissociative responses. Moreover, in NET, survivors of trauma are encouraged to recall the prominent, positive experiences, such as the memories of a caring person or societal success and reward. The intervention calibrates the cognitive networks and develops resources for the survivor.

Procedurally in NET, the survivor chronologically constructs a life story. Empathic understanding, active listening, congruency, and unconditional positive regard are key components of the therapist's behavior. For traumatic stress experiences, the therapist asks in detail for sensory memories, cognitions, emotions, and physiological responses. While narrating, the survivor is encouraged to relive traumatic experiences with all of the various emotional responses while simultaneously maintaining the connection to the “here and now.” Reminding the survivor that the current feelings and physiological responses result from the recall of memories, the therapist links these to autobiographic context, that is, to where and when the event occurred. The therapist is supportive yet directive in the elicitation of the narrative in order to counter avoidance and recover the full implicit information of the trauma.

The documented testimonial biography offered to the survivor following treatment has proven to be a major incentive to complete treatment, rendering drop-outs rare. The effectiveness of NET has been demonstrated with remarkable improvements in trauma-related symptomatology, psychosocial functioning, and physical health (Stenmark, Catani, Neuner, Elbert, & Holen, 2013).

This evidence supports the relevance of *exposure including emotional reliving* for successful treatment. Exposure addresses the challenges of separating the here and now from the there and then. Moreover, NET organically makes meaning of the highly stressful events having occurred during the life span. The respective cognitive work—*meaning making* within NET—provides an essential ingredient for healing. Finally, the processing of positive experiences mobilizes the *resources* a survivor may have and incentivizes the continuation of therapy.

Recent evidence sheds light on the roles of social acknowledgement, social status, and social emotions in the failure to respond to treatments. Therefore, violent acts committed during civilian life or on military duty need to be processed during therapy, paying particular focus to acts perpetrated that break social norms. Crimes committed, guilt, and shame need to be addressed before ex-combatants or criminal offenders may begin the reintegration process into society. In NET, the positive events and the potentially traumatizing, negative events are explored and can be accompanied by combat events, which include not just aversive experiences but also positive moments such as victory, satisfaction of appetitive aggression, or even combat high (Elbert, Weierstall, & Schauer, 2010). We have demonstrated the disseminability of this NET version and its effectiveness to simultaneously reduce criminal acts and trauma symptoms (Crombach & Elbert, 2015), but more work is needed to reintegrate former members of armed groups into a peaceful society (Hermenau, Hecker, Maedl, Schauer, & Elbert, 2013). Especially, social support and negative social interactions may promote or prevent the development of PTSD and delinquent behavior. Therefore, social acknowledgement and recognition of the traumatic experiences and also of the positive feelings experienced during combat may offer a clue for future interventions.

## Edna B. Foa (Prolonged Exposure Therapy)

Prolonged Exposure Therapy (PE) consists of four components, two of which are principal. The first is repeated revisiting and recounting of distressing trauma memories (imaginal exposure) that are avoided because they cause pain, and for many PTSD sufferers they are perceived as leading to “losing control.” Imaginal exposure is followed by 15–20 minutes of processing (discussing the imaginal exposure experience, changes in perceptions that might occur as a result of the experience, and other related emotions and perceptions). The discussions during processing focus not only on fear and anxiety but also on shame, guilt, and anger. A dismantling study (Bryant et al., 2008) showed that excluding postexposure processing from PE results in inferior outcomes, suggesting that conducting processing is important. The second principal component is gradually approaching avoided, safe trauma-related situations (*in vivo* exposure). Dismantling studies indicate that both components contribute to the efficacy of PE. The contribution of education and breathing remains unknown.

PE is based on emotional processing theory (EPT; Foa & Kozak, 1986) and its adaptation to PTSD (Foa & Cahill, 2001) which posits that the erroneous cognitions of “the world is extremely dangerous” and “I am extremely incompetent” mediate the development and maintenance of PTSD by promoting avoidance that prevents the individual from disconfirming these cognitions. In PE, disconfirmation of negative cognitions occurs via imaginal exposure, processing, and *in vivo* exposure. Several studies support this assertion (Foa, Tolin, Ehlers, Clark, & Orsillo, 1999; Kleim et al., 2013). Moreover, reductions in negative cognitions precede decreases in PTSD symptoms (Zalta et al., 2014), suggesting that such reductions constitute a mechanism underlying PTSD symptom reduction during PE and other cognitive behavioral treatments. EPT posits that two conditions are required for successful treatment, both empirically validated: (1) activation (emotional engagement) of the trauma memory and (2) the presence of information that disconfirms expected harm during exposures (i.e., disconfirmation of negative expectations). As noted above, many experts share the view that reduction in negative cognitions is an active mechanism in treatment for PTSD. PE emphasizes activation (emotional engagement) of the trauma memory during treatment as an additional active mechanism of treatment. Data from animal studies support activation as a treatment mechanism; indeed, lack of fear activation prevents fear extinction (Gillihan & Foa, 2011).

Where do we go from here? As is clearly indicated in this paper, we have several evidence-based treatments for PTSD with similar efficacy. However, treatments differ in the strength of evidence for their efficacy. Also, treatments differ in terms of the knowledge of which treatment components are active and which are not. More studies need to examine the relative contribution of different components to the efficacy of treatment, and thus streamline treatment. One important issue in the field is: are interventions that enhance emotion regulation needed to enhance treatment designed to decrease PTSD (Minnen, Harned, Zoellner, & Mills, 2012)? Studies have shown that emotion regulation is improved via skills training (Cloitre, Cohen, Koenen, & Han, 2002). However, Cloitre and colleagues presented data that suggest that prolonged exposure, in and of itself, leads to improvement in emotion regulation (Cloitre et al., 2010). Further research is necessary to explore emotion regulation as a mechanism of action in treatment. The causal impact of change in negative cognitions on PTSD reeducation should also be further studied, using measures other than self-report. Another line of research that requires further examination is the use of extinction enhancers to augment exposure treatments with the goal of increasing treatment efficacy and efficiency (Hendriks, De Kleine, & Van Minnen, 2015). Finally, and most important, is the study of effective ways to disseminate and implement our evidence-based treatments into community clinics around the world.

## Berthold P. R. Gersons (Brief Eclectic Psychotherapy for PTSD)

Brief Eclectic Psychotherapy for PTSD (BEPP) is one of the evidence-based, effective trauma-focused treatments (Gersons, Meewisse, & Nijdam, 2015; Nijdam, Gersons, Reitsma, De Jongh, & Olff, 2012). It combines five modules from different origins. It starts with *psychoeducation* together with a partner or trusted person. The connection between the PTSD symptoms and the traumatic event(s) is explained and understood. Then, the treatment will be explained. The next four to six sessions of a total of 16 are used for *imaginal exposure*. In BEPP, the imaginal exposure is a very slow but detailed process, starting with a relaxation exercise, then focusing on the period shortly before the traumatic event, the event itself and the follow-up. It is focused on the expression of emotions during the hotspots (Nijdam, Baas, Olff, & Gersons, 2013) and not on habituation of the fear response. For instance, when someone survives an airplane crash, it starts with the trembling of the plane, then the crash, the crumbling of the cabin, dying people in the cabin, climbing out a hole, being hurt, and then trying to reach a safe place outside, etc. Other tools are the use of *memorabilia* connected to the traumatic events and the writing of an *ongoing letter* to express emotions of anger or also grief. The last nine sessions are devoted to giving meaning and learning from the traumatic event (Gersons & Schnyder, 2013). Treatment ends with a farewell ritual. The BEPP-protocol is now available in eight languages (Dutch, English, German, Georgian, Italian, Lithuanian, Polish, and Spanish) and is being modified for children and for traumatic grief.

For successful treatment of PTSD, three key elements can be identified. The first is the patient can trust the therapist to be a non-judgmental, empathic listener to the awful experiences of the past. Second, this must help the patient to relive in exposure the events in a safe environment where the connected emotions of grief, sorrow, and anger can be expressed freely. Third, however, different words are used for this, is learning from the experience how life can be endangered and dangerous, making it worthwhile to enjoy life anew.

It is good to realize we are still standing at the beginning of successful treatment of PTSD. One important theme to pay attention to is to get a better view of which symptoms fully disappear after treatment and which residual symptoms will stay. It seems as if always a “pilot flame” of vulnerability to new traumatic events will stay after treatment (Gersons & Olff, 2005). A recent evaluation of the Dutch police outpatient department reported that 96% of 566 police officers no longer fulfilled the PTSD diagnosis after BEPP treatment (Smit et al., 2013). However, 60% showed still minor symptoms of concentration problems after treatment. A second topic is the fact that while evidence-based treatments of PTSD have the same effect size, every treatment modality even using the same key words has specific different protocolized ways to reach the positive results. There is a need to streamline in developing an overarching protocol for treating PTSD. Meanwhile, a third emerging theme should be to recognize the different needs of very different patient groups regarding age, culture, sex, and differences in experiences. An example is to focus more on traumatic grief in treatment of PTSD instead of restricting it to the decrease of fear. Also, skills training is an example of recognizing other needs in treatment of our patients.

## Patricia A. Resick (Cognitive Processing Therapy)

My first thought about commonalities was that all evidence-based treatments for PTSD include psychoeducation and a focus on traumatic events for change in emotions, cognitions, and avoidance. When I first developed Cognitive Processing Therapy (CPT) (Resick & Schnicke, 1992), I thought key elements of treatment included education, cognitive therapy around erroneous beliefs about the trauma, and a trauma account to encourage emotional expression and the need for the therapist to understand the details of the trauma. However, when I conducted a dismantling study of CPT with and without the written accounts, I not only found graphic accounts did not add anything to the protocol but also slowed the progress of therapy. The CPT cognitive-only version (CPT-C) achieved clinically meaningful improvement by the fourth session through focused Socratic dialogue. CPT with accounts postponed improvement until after the accounts were completed (Resick et al., 2008). Since then, my own research has been conducted with CPT-C. One might argue that any discussion about traumas constitutes exposure to avoided memory. However, there is a difference between talking *about* why something happened and reexperiencing the memory of the trauma in graphic detail.

Then, I thought about therapies such as present-centered therapy (PCT) that were supposed to be control conditions for other studies that do not focus on the traumatic events at all, but educates about PTSD and then focuses on problem solving current symptoms and issues. A recent meta-analysis of five trials that included PCT as a control condition (Frost, Laska, & Wampold, 2014) found small effect size differences between PCT and the active PTSD treatment and large effect size differences between PCT and waiting list (0.74–1.27). PCT also had lower drop-out rates than the trauma-focused treatments. Of course, one might consider that some people drop out of treatment because they are doing well, and some people stay in treatment because they are not improving, so drop-out may not be as important as once thought (Szafranski, Smith, Gros, & Resick, in preparation). The meta-analysis did not examine the effects of PCT over time, but given initial findings, we have to consider the mechanisms of change when there is no discussion of the trauma memories, and the focus is on symptoms and current problems.

One thing all treatments have in common is *education* of clients about PTSD and about different ways to *think* about their problems, past or present. Any intervention that actively engages the client's prefrontal cortex is, because of the reciprocal relationship with the amygdala, going to teach *affect regulation* and is going to be calming. We also should not underestimate the *non-specific effects of treatment*. By this I do not mean placebo effects, but the very real effects of entering therapy, taking time out from one's day and the costs involved, focusing on one's problems, discussing them with an empathetic and skilled therapist, and leaving with a plan of action. Clients who engage in therapy have made an investment in their well-being. Most therapies whether evidence-based or not are probably going to improve functioning in clients with PTSD to some extent. It may be one reason why there is reluctance for many therapists to try evidence-based treatment protocols. They believe, and probably rightly so, that their clients have improved. The question is whether we can do better than these non-specific effects and education. For that we need large enough trials that are powered for medium to small effect sizes and to keep working on refining our therapies until they provide better outcomes than generic therapy and specifically with comorbid conditions.

## Francine Shapiro (EMDR Therapy)

Eye Movement Desensitization and Reprocessing (EMDR) therapy is a comprehensive eight-phase approach emphasizing the roles of memory and the information processing system in the origin and treatment of psychopathology (Shapiro, 2001, 2014b). It is posited that unprocessed memories of adverse life experiences, which include the emotions, beliefs, and physical sensations experienced at the time of the event, are stored inappropriately in episodic memory and underlie current dysfunctional responses. EMDR processing of the event facilitates connections to integrated semantic memory networks that provide corrective information, resulting in the internal generation of insights, changes to appropriate emotions, and the emergence of a coherent narrative. Education about the nature of pathology and specific affect-change techniques are provided to ensure a sense of empowerment during and between the sessions. Clients are not asked to describe the memory in detail but rather focus initially on an image of the event, the currently held negative belief, and location of disturbing body sensations. Processing involves short exposures of approximately 30 s, paired with sequential sets of bilateral eye movements that cause significant decreases in arousal, negative affect, and imagery vividness (Lee & Cuijpers, 2013). Clients are instructed to “let whatever happens, happen” as new thoughts, emotions, sensations, or memories generally emerge. After each set, they are asked to briefly report what comes to mind, and the clinician guides their focus of attention for the next set according to standardized protocols. The processing procedures facilitate and evaluate changes in affective, cognitive, and somatic responses, until the memory is resolved. Processing of the memory is generally completed within one to three sessions. Overall, treatment includes processing past memories, present triggers, and future challenges.

Three elements are crucial to treatment: (1) providing clinical experiences and techniques to ensure stabilization and a sense of self-mastery, (2) processing memories and triggers, and (3) teaching skills needed for appropriate social interactions. Successfully treated clients are able to modulate their responses and demonstrate adaptive functioning in challenging situations. Clients who were multiply abused in childhood can benefit from more extensive education and experiences that increase access to positive memory networks (Korn & Leeds, 2002; Shapiro, 2001). Interactions within the therapeutic relationship may provide clients with their first opportunity to discover that they are of value and worthy of unconditional regard. Lasting clinical effects are derived from processing memories of adverse experiences and current triggers (Shapiro, 2014b; Solomon & Shapiro, 2012). Although effective treatment of an individual memory can generalize to associated events, current situations should be assessed for the effects of second-order conditioning and processed accordingly. Comprehensive assessment and incorporation of skills needed for the future are vital. The time needed for skill acquisition is determined by whether it is necessary to address developmental deficits due to the lack of appropriate socialization experiences during childhood. The goal for all clients is adaptive functioning, both individually and relationally.

Future developments should take into account important emerging themes, including “moral injury” and the impact of accumulating adverse life events. Processing with EMDR therapy transmutes guilt and shame to acceptance, which is often verbalized as “I did what I had to do” (Russell & Figley, 2012). The short exposures used are posited to result in reconsolidation, whereby the original memory is stored in altered form as a source of resilience (Shapiro, 2014b; Solomon & Shapiro, 2012). Comprehensive evaluation of the full clinical picture should identify any continued areas of disturbance and associated memories of adverse experiences that should be processed to resolution (Shapiro, 2001, 2014b). Some unanswered questions involve the investigation of diverse trauma-related conditions. Rigorous research should explore further the usefulness of trauma memory processing as a treatment for conditions traditionally considered intractable. Examples include chronic phantom limb pain (De Roos et al., 2010), deviant arousal (Ricci, Clayton, & Shapiro, 2006), and psychotic symptoms (Van den Berg & Van den Gaag, 2012). In addition, the negative psychological, physical, and societal effects of trauma and other adverse experiences have been clearly demonstrated (Shapiro, 2014a), even indicating “multiple risk factors for several of the leading causes of death in adults” (Felitti et al., 1998). These findings underscore the need for future research to determine the best ways to destigmatize mental health treatment and increase the utilization of effective intervention programs worldwide.

## Conclusions

The currently available empirically supported psychotherapies for trauma survivors have a lot in common. Commonalities identified by contributors include:

1. *Psychoeducation* offers information on the nature and course of posttraumatic stress reactions, identifies ways to cope with trauma reminders, and discusses strategies to manage distress. In trauma-focused psychotherapy, psychoeducation aims at facilitating interventions, optimizing patient cooperation, and preventing relapse.
2. *Emotion regulation and coping skills* are frequently taught and trained across many therapeutic approaches. In some instances, this is done more implicitly, in others as an explicit element of the treatment.
3. *Imaginal exposure* is strongly emphasized in PE and NET. However, some form of exposure to the patients’ memory of their traumatic experiences can be found in virtually all evidence-based psychotherapies for trauma-related disorders.
4. *Cognitive processing, restructuring, and/or meaning making* is another element that can be found in almost all of the empirically supported psychological treatments for PTSD. Although, in the cognitive approaches, these are the most important treatment components, in other protocols, they are conceptualized as part of the integration that takes place after or during exposure.
5. *Emotions* are targeted in all psychotherapies. Some predominantly tackle the patients’ trauma or fear network, others focus more or equally on guilt and shame, anger, or grief and sadness.
6. *Memory processes* also play an important role in treating trauma-related disorders. No matter which technical terms are used, the reorganization of memory functions and the creation of a coherent trauma narrative appear to be central goals of all trauma-focused treatments.

Regarding future directions for research, many of us proposed that attention should be given to the issue of *post-treatment residual symptoms and vulnerability* to new traumatic events.

A better understanding of underlying *mechanisms of action* is clearly needed. Such systematic research can help identify the most effective treatment elements, so that therapies can become more powerful and more streamlined. In addition, studying mechanisms can also help identify processes or mechanisms that have been overlooked and that may significantly affect outcome. Candidates for study include changes in cognition and cognitive processes (e.g., increased ability to discriminate old and new memories), and in emotion regulation (e.g., ability to self-soothe, tolerate distress, recognize and accept the presence of conflicting or opposing emotions). Novel mechanisms for consideration may include the role of more “social” emotions, cognitions, and behaviors, such as attachment and social bonding processes, empathy, and compassion (therapeutic alliance), as well as the opposite experiences of social distance and social rejection (being an “outcast”) and associated experiences of moral injury.

We would also recommend developing treatments that are *tailored to the needs of different patient groups* with regard to factors such as age, sex, culture, comorbidities, and type of trauma experience. The latter include, for example, understanding the neurobiological and psychosocial effects of chronic and multiple traumas, particularly those during the various developmental phases, and experiences of perpetrating acts of violence or those inconsistent with one's moral stand (e.g., “moral injury”).

We recognize the need to think more broadly about the *continuum of trauma care* which ranges from primary prevention to strategies for posttrauma reintegration. This includes creating prevention programs for high-risk populations (e.g., military or emergency personnel) that strengthen resiliency, and programs that facilitate social acknowledgement of the experiences of trauma populations as they reenter the flow of everyday life. Some of us advocate for future directions in which interventions are made brief and shorter still, whereas others endorse longer and multifaceted therapies that introduce the social context as an integral part of treatment (e.g., systems interventions). While there is likely to be uniform recognition of social and political history as part of the trauma and recovery process, the implications for organizing the frame for psychotherapy (e.g., do we treat the individual, family, or community?) and how it might differ by history and culture remain unknown and deserve attention.

Finally, as a caveat, we reflect on the fact that we all come from different countries and that our patients also vary quite a lot with regard to ethnicity, culture, and personal history (Schnyder, 2013). We treat patients not diagnoses, thus while we share a common language and common terms, there may be unidentified gaps and mismatches in what we mean when we speak about the clinical phenomena (e.g., negative cognitions or emotional engagement), the interventions, and the nature of the patient response. We may fall short of identifying important differences in the particulars of the content and delivery of the interventions as well as the patient's responses that are driven by culture and context-specific values and the stories they have to tell.

Even with these limitations in mind, the therapies reviewed in this article—each with its different focus—have all been shown to be effective, providing clinicians with an array of empirically supported treatment choices to benefit their patients. We hope that the common elements identified in this article as critical in treating PTSD will serve as a guide to future development, supporting clinicians in their ongoing attempts to provide the best possible trauma-focused psychotherapy to their patients.

## Footnotes

1 We are grateful to Dr. Richard Bryant who, at the end of the panel, asked the panelists to identify three key treatment interventions that are common and critical in treating PTSD.
