# Corrigendum

**DOI:** 10.3402/ejpt.v6.29481

**Published:** 2015-08-31

Regarding the Invited Review Article ‘Psychotherapies for PTSD: what do they have in common?’ by Ulrich Schnyder et al.

Published in European Journal of Psychotraumatology 2015. Citation: European Journal of Psychotraumatology 2015, **6**: 28186 - http://dx.doi.org/10.3402/ejpt.v6.28186

In the paragraph headed “Thomas Elbert, Maggie Schauer, and Frank Neunert (Narrative Exposure Therapy)” one of the names were incorrectly spelt.

The correct name is Frank Neuner (not Frank Neunert). The correct heading is displayed below.

Thomas Elbert, Maggie Schauer, and Frank Neuner (Narrative Exposure Therapy)

